# Case Report: Submucosal gastroblastoma with a novel *PTCH1::GLI2* gene fusion in a 58-year-old man

**DOI:** 10.3389/fonc.2022.935914

**Published:** 2022-09-06

**Authors:** Cuimin Chen, Junliang Lu, Huanwen Wu

**Affiliations:** ^1^ Department of Pathology, Shenzhen Hospital of Peking University, Shenzhen, China; ^2^ Department of Pathology, Peking Union Medical College Hospital, Peking, China

**Keywords:** gastroblastoma, *PTCH1::GLI2* fusion, stomach, GLI1, GLI2

## Abstract

Gastroblastoma is a rare biphasic tumor of the stomach that generally presents in young patients. *MALAT1*-*GLI1* gene fusion was considered to be the characteristic molecular alteration of this tumor in previous reports. Herein, we described a 58-year-old man with a mass mainly located in the submucosa of the stomach. Microscopic examination showed a biphasic morphology with the same immunohistochemical phenotype as gastroblastoma. Interestingly, a novel *PTCH1::GLI2* fusion rather than *MALAT1-GLI1* fusion was detected in the tumor by RNA-based next generation sequencing (NGS). This was the first report that demonstrated a novel *PTCH1::GLI2* gene fusion in gastroblastoma, and thus expanded the molecular spectrum of this tumor. The underlying pathogenesis merits further investigation.

## Introduction

Gastroblastoma, first reported by Miettinen et al. in 2009 (1), is an extremely rare tumor of the stomach with biphasic morphology. It typically affects young people with potential for local recurrences and distant metastases. Graham and colleagues revealed recurrent somatic MALAT1-GLI1 gene fusions in their 4 cases of gastroblastoma in 2017 (2). Thereafter, Castri et al. reported the fifth case of gastroblastoma with identical MALAT1-GLI1 fusion in an elderly patient (3). According to the 2019 World Health Organization (WHO) classification of tumors of the digestive system, both biphasic histology and MALAT1-GLI1 gene fusion are essential for the diagnosis of gastroblastoma (4). Recently, a novel EWSR1-CTBP1 fusion was identified in a gastric tumor morphologically consistent with gastroblastoma in a patient with Wiskott-Aldrich syndrome (5). Herein, we report an unusual case of a 58-year-old man with a biphasic tumor in the submucosa of the gastric body. The morphology and immunoprofiles suggested a diagnosis of gastroblastoma. However, a novel *PTCH1::GLI2* gene fusion was found in the tumor cells, instead of the previous reported characteristic MALAT1-GLI1 gene fusion or recent described EWSR1-CTBP1 fusion in gastroblastomas. Thus, our finding expands the molecular spectrum of this extremely rare tumor.

## Case presentation

An asymptomatic gastric mass was accidentally found in a 58-year-old man during a routine check-up. The endoscopic ultrasound was performed and a submucosal protuberant mass measuring 1.98 X 1.86cm was detected on the lesser curvature of the gastric body. No treatment was performed at that time. Two months later, repeated endoscopic ultrasound revealed that the mass increased to 2.43 X 1.47cm and showed a hemispherical bulge appearance in the stomach with central ulceration. The mass was uniformly hypoechoic. Enhanced computed tomography scan showed a submucosal mass in the posterior wall of the stomach with suspicion of glomus tumor. The patient underwent endoscopic submucosal dissection.

**Figure 1:**
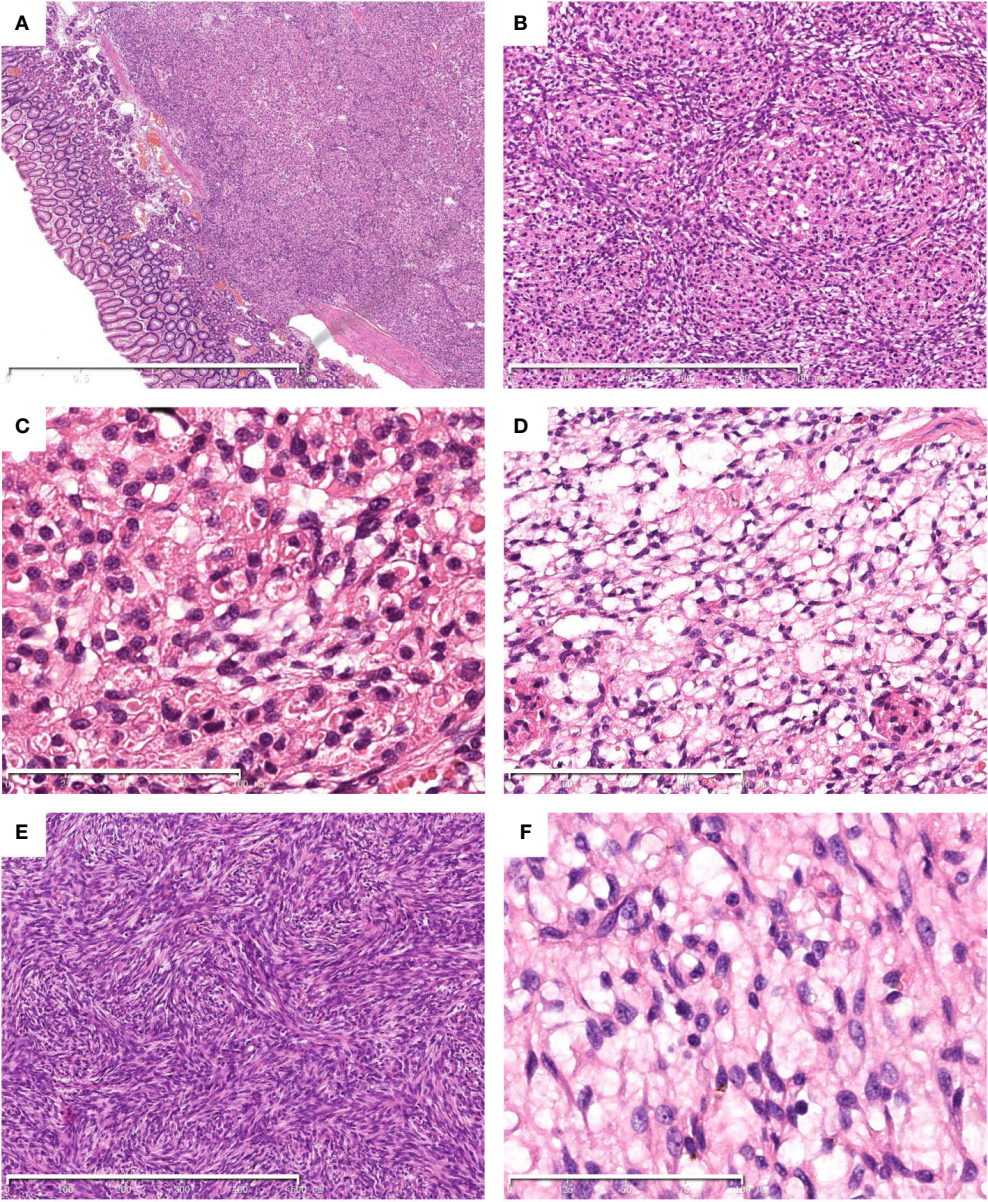
Histological findings of the tumor. Hematoxylin and eosin (HE) stain. The tumor centered in the submucosa with focal invasion to the mucosa. **(A)** A biphasic histology with epithelial and spindle cells was noted **(B)**. Cytoplasmic eosinophilic granules and globules were observed in the bland-looking epithelial cell component **(C)**. The spindle cells arranged in reticular **(D)** and storiform **(E)** pattern. The spindle cell component shows a bland appearance **(F)**. The scale bars (2mm in **A**, 500μm in **B** and **E**, 200μm in **D**, 100μm in **C** and **F**).

Histological examination showed a well-demarcated tumor centered in the submucosa with focal extension to the mucosa (**Figure 1A**) with ulcer formation. The tumor showed a biphasic morphology, consisting of relatively uniform epithelial cells and spindle cells (**Figure 1B**). The epithelial cell component was arranged in nests, sheets, clusters, and cords with small round to oval nuclei with fine chromatin, inconspicuous nucleoli and scant to moderate eosinophilic cytoplasm. Distinctive eosinophilic granules and globules were also observed in the cytoplasm (**Figure 1C**). The spindle cell component had hypocellular myxoid areas with a reticular growth pattern (**Figure 1D**) and hypercellular areas with fascicular and storiform growth patterns (**Figure 1E**). The spindle cells had pale eosinophilic cytoplasm and elongated or plump nuclei with or without small nucleoli (**Figure 1F**). Mitotic figures were infrequent and necrosis was absent.

**Figure 2:**
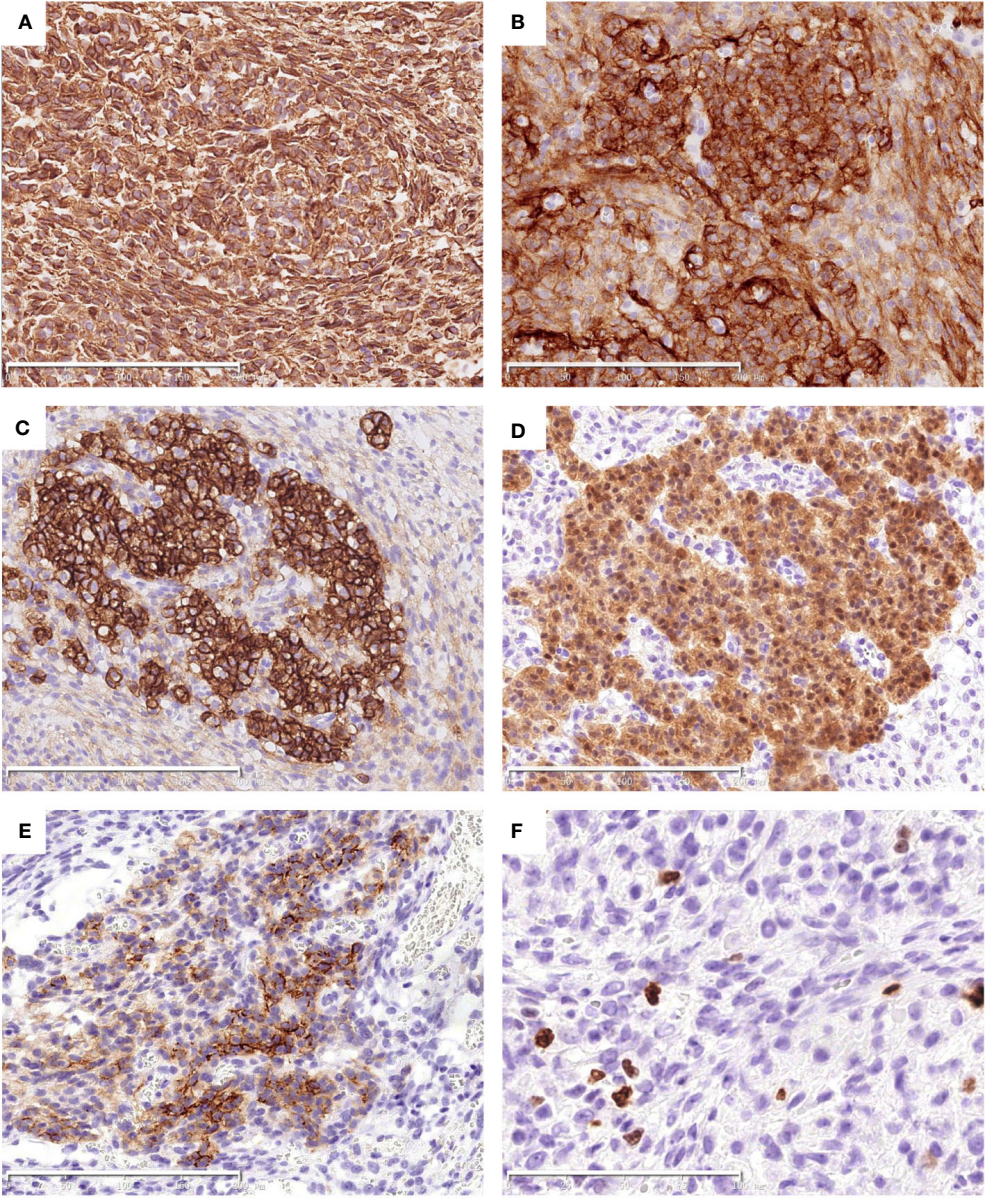
Immunohistochemical findings of the tumor. Both the epithelial and spindle cell components diffusely express Vimentin **(A)** and CD10 **(B)**. The epithelial cells are strongly positive for CD56 **(C)** and S100 **(D)**, and focally positive for EMA **(E)**. The Ki-67 index was low **(F)**. The scale bars (200μm in **A–E**, 100μm in **F**).

By immunohistochemistry, both the epithelial and spindle cell components were diffusely positive for vimentin (**Figure 2A**), CD10 (**Figure 2B**) and bcl-2. The epithelial cells also strongly expressed CD56 (**Figure 2C**) and S100 (**Figure 2D**), and showed focal staining for EMA (**Figure 2E**), whereas the spindle cell component lacked expression of CD56, S100 and EMA (**Figures 2C–E**). The Ki-67 proliferative index was low (about 5%) (**Figure 2F**). CD117, DOG1, CD34, SMA, desmin, SOX10, CD99, chromogranin A, synaptophysin, CAM5.2, and AE1/AE3 were negative. SDHB was preserved in both components.

**Figure 3:**
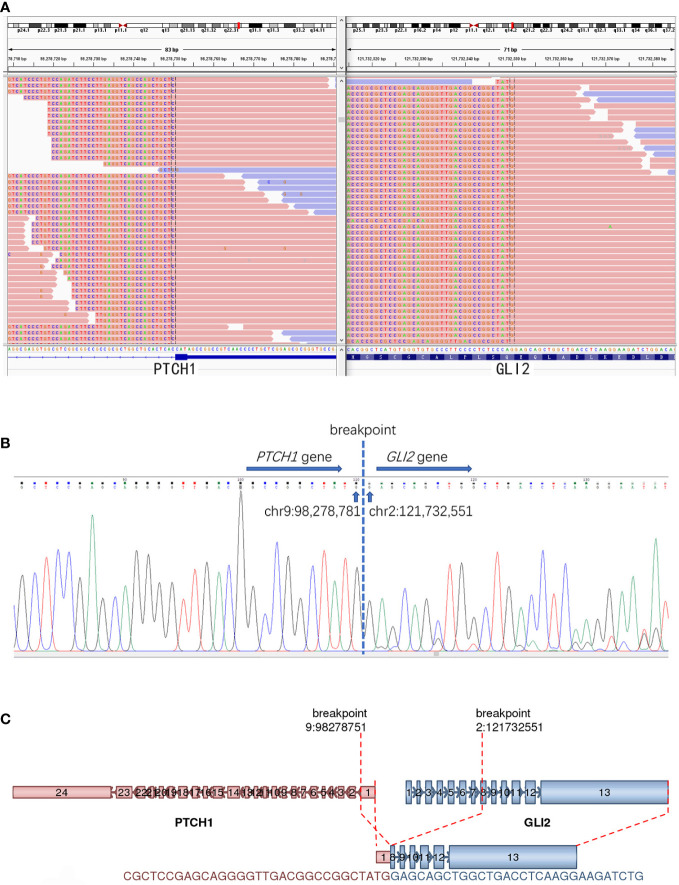
The detection of PTCH1::GLI2 fusion. **(A)** RNA-based NGS revealed a novel PTCH1::GLI2 fusion. **(B)** PTCH1::GLI2 fusion were confirmed by Sanger sequencing. **(C)** Schematic representation indicates PTCH1 (exon 1) ::GLI2(exon 8) fusion. Arrows indicate the direction of transcription for each gene. Exons and introns are represented by colored boxes and lines, respectively.

RNA-sequencing was used to detect known and novel fusion genes across the whole transcriptome. The detailed methods were described in our previous study (6). It revealed a novel *PTCH1*(exon 1)::*GLI2*(exon 8) fusion, which was further confirmed by Sanger sequencing (**Figures 3A–C**). The 5′ of gene *PTCH1* (exon 1) was fused to the 3′ of gene *GLI2*(exon 8).

## Discussion and conclusions

Gastroblastoma was first described by Miettinen and colleagues in 2009 as a biphasic gastric tumor with variable proportions of epithelial and spindle cells. The reported cases of gastroblastoma had a wide age range at diagnosis, from 9 to 74 years (3, 7, 8), although most cases occurred in young adults. There was no apparent gender predilection. Some patients had local recurrence or developed lymph node or distant metastases (2, 3, 9, 10). However, most reported cases had no evidence of disease during follow-up after surgical resection. Given the rarity of this tumor, the biological behavior is still unclear and the optimal therapeutic strategy has not been established.

Gastroblastoma usually arise in the gastric muscularis propria and may extend to the subserosa, submucosa and mucosa. Histologically, the tumor shows a biphasic histology characterized by uniform epithelial cells and spindle cells component without obvious atypia or pleomorphism. The epithelial cells may arrange in nests, cords, tubules, clusters and sheets pattern with scant pale eosinophilic cytoplasm, round nuclei, and inconspicuous nucleoli. Rosette-like structures with central eosinophilic secretions are frequently present. However, no rosette-like structures or tubules were noted in our case. In addition, eosinophilic granules and globules were easily found in the cytoplasm of the epithelioid cells in our case, which have not been reported previously (1, 2, 4, 8). The spindle cell component is monotonous and characterized by bland, elongated, slender cells arranged in reticular, fascicles or sheets pattern, often in a myxoid background. Mitotic figures are rare in most cases (4). In our case, storiform growth pattern was present in some areas as well (**Figure 1E**). Immunohistochemically, the epithelial cells show variable expression of keratins, EMA, CD10, and CD56, while the spindle cells lack keratins immunoreactivity, but instead show CD10 and vimentin positivity, with or without CD56 expression. CD117/KIT, DOG1, CD34, SMA, desmin, chromogranin, synaptophysin, CD99 and CDX2 are generally negative (2, 8). The Ki-67 index is usually low. Unlike the previously reported cases, the epithelial cells in the present case were strongly positive for S100. The eosinophilic granules and globules, as well as S100 immunoreactivity in the epithelial component have not been reported previously, and may be associated with *PTCH1::GLI2* fusion in our case. However, it requires further verification in more cases. Other gastric neoplasms with biphasic histology should be in the list of differential diagnosis of the present case. Primary synovial sarcoma (SS) of the digestive system has been reported in a few literatures (11). However, most reported gastric synovial sarcomas were classified as monophasic subtype, while only an extremely small number of cases shows a biphasic morphology. Immunoprofiles overlap may present between SS and gastroblastoma. The absence of specific *SS18* gene rearrangement is helpful to rule out SS. In our case, RNA-based NGS revealed *PTCH1::GLI2* fusion rather than SS18 rearrangement, excluding the diagnosis of SS. Gastrointestinal stromal tumors (GISTs) are the most common mesenchymal neoplasms of the stomach. Some GIST cases may show a biphasic epithelial/spindle cell histology. Negativity for CD34, CD117, and DOG1 by immunohistochemistry can eliminate this diagnosis. In our case, the positive staining for SDHB ruled out the possibility of SDH-deficient GIST as well. Nevertheless, some gastroblastoma cases may show positive CD117 immunoreactivity (7, 9). Molecular analysis of KIT and PDGFRA mutations, which can be found in the majority of GISTs, is essential in difficult cases. Malignant gastrointestinal neuroectodermal tumors (GNETs) occur mainly in the wall of gastrointestinal tract. Some GNETs cases display a biphasic morphology composed of epithelioid and spindle cells arranged in solid sheets, nests, papillary, pseudoalveolar, microcystic and fascicular pattern (12, 13). In contrast to GNETs, gastroblastomas usually express keratins and EMA. In addition, gastroblastoma lacks diverse growth patterns and characteristic EWSR1-ATF1/CREB1 fusion of GNETs. GNETs generally show strong and diffuse S100 and SOX10 positivity. In our case, the epithelial component showed S100 positivity, but lacked expression of SOX10. Furthermore, RNA-based NGS revealed no EWSR1-ATF1/CREB1 fusion in our case, ruling out the diagnosis of GNET. Other tumors with biphasic morphology should be considered in the differential diagnosis, such as carcinosarcoma, mesothelioma, and clear cell sarcoma. Comprehensive evaluation of the tumor location, morphology, immunohistochemistry, and molecular analysis can contribute to distinguish these entities.

Recurrent somatic MALAT1-GLI1 fusions in gastroblastoma were first identified by Graham and colleagues in their series of four cases in 2017 (2). Subsequently, Castri et al. found the same molecular change in another case of gastroblastoma (3). Nevertheless, molecular detection has not been carried out in a certain number of other previous reported cases, so the actual number of gastroblastoma carrying MALAT1-GLI1 fusion is still unknown. Most recently, a novel EWSR1-CTBP1 fusion in the gastroblastoma tumor cells has been reported in a patient with pre-existing Wiskott-Aldrich syndrome (5). Another case of a duodenal biphasic tumor sharing similar clinicopathological features with gastroblastoma, termed “Duodenoblastoma”, although exhibiting somewhat different immunoprofiles (CD10 negativity and desmin, CD99, SMA, progesterone receptor positivity in mesenchymal component), has been reported, suggesting a possible extragastric location of such type of tumors (14). A recent case of jejunal malignant neoplasm with MALAT1-GLI1 fusion has also been reported, but the primary tumor displayed a high-grade appearance with markedly atypical epithelioid and spindle cells and multiple necrosis, which made it different from the classic gastroblastomas reported before. Antonescu et al. described a series of six malignant neoplasms harboring GLI1 gene rearrangement in 2019, which mainly occurred in the soft tissue (except one in the bone), and put this novel pathologic entity under the descriptive terminology of “malignant epithelioid neoplasms with GLI1 fusions” (15). The cohort of these tumors were composed of bland-looking monomorphic epithelioid cells arranged in nests and cords with intricate capillary network, and some tumors (4 of 6 cases) expressed S100 protein. All 6 cases showed recurrent GLI1 fusions with either ACTB1, MALAT1, or PTCH1. In our case, the tumor also positively expressed S100 in the epithelial component, but showed a biphasic histologic resemblance to gastroblastoma, while no areas of spindle cells were noted in the above “malignant epithelioid neoplasms with GLI1 fusions”.

In our case, a novel *PTCH1::GLI2* fusion was detected in the tumor cells, instead of the previous reported characteristic MALAT1-GLI1 fusion in gastroblastomas. PTCH1 and GLI2 are two members of Hedgehog (Hh) signaling pathway. Hh ligands bind to the transmembrane protein receptors Patched (PTCH, including PTCH1 and PTCH2) and lift the inhibition of smoothened (SMO) by PTCH, then allowing the transduction of the signal, eventually leading to the activation of GLI which induces the upregulation or downregulation of multiple downstream targets. Hh pathway plays an important role in embryonic development and aberrant activation of Hh pathway may lead to congenital abnormalities as well as tumorigenesis (16).

The glioma-associated oncogene homologue (GLI) family consists of GLI1, GLI2 and GLI3, all of which have a highly conserved zinc finger domain and act as important transcription factors in the Hh pathway. GLI1 has been reported to function in variable tumors (17). Among them, several GLI1 fusion associated tumors have been reported in recent years. MALAT1-GLI1 fusion was identified in some plexiform fibromyxomas and gastroblastomas. The gene fusion causes overexpression of GLI1 mRNA and protein, thus leading to the activation of its downstream targets, which may contribute to tumorigenesis. ACTB-GLI1 fusion have also been detected in some cases known as “pericytoma with (7;12) translocation” (18). The fusion of GLI1 with the β-actin gene (ACTB) leads to the replacement of the promoter region of GLI1 with that of the ubiquitously expressed ACTB gene, resulting in the overexpression of GLI1 sequences, thus cause deregulation of the GLI1 target genes. In contrast to the oncogenic function of GLI1 which have been reported in many types of tumors, there were relatively few studies on that of GLI2. Increased expression of GLI2 has been reported to promote the development of osteosarcoma, breast cancer and pancreatic tumors (19–21). Some recent studies showed that upregulation of GLI1 and GLI2 was observed in basal cell carcinoma (BCC) and GLI2 could directly activate GLI1 expression by binding to its promoter (22). Interaction of GLI1 and GLI2 was found in pancreatic cancer cells as well (21).

PTCH1 is one of the Hh receptor genes as mentioned above. Germline mutations of PTCH1, such as nonsense mutations and frameshift mutation, can give rise to Gorlin syndrome. PTCH1 mutation has also been found in sporadic BCC, medulloblastoma, squamous cell carcinoma and rhabdomyosarcoma (23–25). Nevertheless, there are few reports of tumors associated with PTCH1 gene fusion. Only one case of *PTCH1::GLI1* fusion has been reported recently by Antonescu et al. in a malignant epithelioid neoplasm in the submandibular area (15). Our present case is the second case involving PTCH1 gene fusion, while *PTCH1::GLI2* fusion in neoplasms has never been reported before. We hypothesized that this gene fusion may have an effect similar to MALAT1-GLI1 fusion, that is, it activates the Hh signaling pathway and lead to tumorigenesis.

In summary, we described the first gastroblastoma case with a novel *PTCH1::GLI2* fusion in an elder patient. Our finding expands the molecular spectrum of gastroblastoma. The signaling pathway inhibitors targeting GLI transcription factors may have potential therapeutic effect on this tumor (26).

## Data availability statement

The original contributions presented in the study are included in the article/supplementary material. Further inquiries can be directed to the corresponding author.

## Ethics statement

Written informed consent was obtained from the participant for the publication of this case report.

## Author contributions

CC was responsible for literature search, photo preparation and wrote the manuscript. HW contributed to the NGS detection, pathologic diagnosis and revised the manuscript. JL contributed to the NGS detection and the photo preparation. All authors have read and approved the final manuscript.

## Funding

The study was funded by grants from the fund of the ‘San-ming’ Project of Medicine in Shenzhen (no. SZSM201812088).

## Conflict of interest

The authors declare that the research was conducted in the absence of any commercial or financial relationships that could be construed as a potential conflict of interest.

## Publisher’s note

All claims expressed in this article are solely those of the authors and do not necessarily represent those of their affiliated organizations, or those of the publisher, the editors and the reviewers. Any product that may be evaluated in this article, or claim that may be made by its manufacturer, is not guaranteed or endorsed by the publisher.

## Abbreviations

WHO, World Health Organization; SS, synovial sarcoma; GISTs, Gastrointestinal stromal tumors; GNETs, Malignant gastrointestinal neuroectodermal tumors; GLI, glioma-associated oncogene homologue; Hh, Hedgehog; PTCH, Patched; SMO, smoothened; BCC, basal cell carcinoma; HE, Hematoxylin and eosin.
